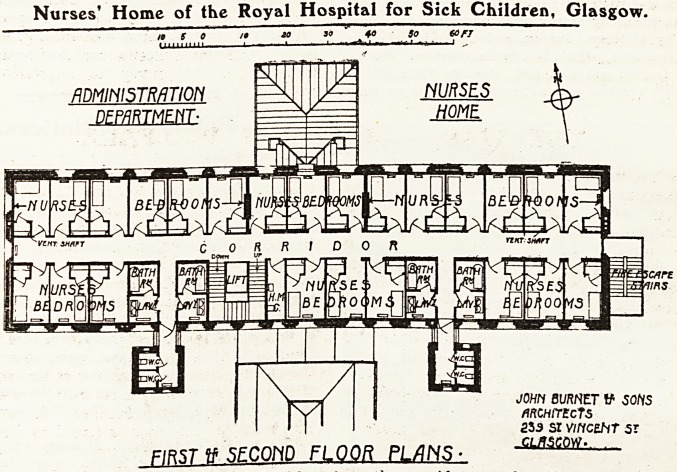# Royal Hospital for Sick Children, Glasgow

**Published:** 1915-09-25

**Authors:** 


### HOSPITAL ARCHITECTURE AND CONSTRUCTION.

### Royal Hospital for Sick Children, Glasgow.

NEW NURSES' HOME.

THIS building forms part of the main administration block (the plan of which, with a detailed description, we published in THE HOSPITAL of July 4, 1914), and is therefore not a self-contained Home. There are three storeys, planned in a similar way to that illustrated. The plan is a quite simple one and consists of a straight corridor, with the rooms arranged on each side. The bedrooms are of the usual type, and each is provided with a fixed cupboard. The bathrooms, of which there are four on each floor, have no direct light or ventilation, a lavatory being in each case interposed between the bathroom and the external wall. On the face of it this is a remarkable arrangement, but possibly some device has been adopted for providing the necessary light and ventilation. The w.c.s are in detached towers connected by cross-ventilated lobbies with the main building.

On the ground floor are sisters' rooms, a drawing-room and a writing-room for nurses, which can be thrown together for special occasions, and a visitors' room.

There is a fire-escape staircase at the east end of the building; the main staircase opens directly on to the corridor, so that if in case of a fire in the basement it were filled with smoke the corridor might be rendered impassable to nurses from the bedrooms at the west end. To make the one escape staircase really effective the inside staircase should have been shut off with a fire-resisting screen and self-closing doors.

The architects are Sir John Burnet and Sons.

**Nurses' Home of the Royal Hospital for Sick Children, Glasgow.**

## Figures and Tables

**Figure f1:**